# HPV Vaccination Completion Among Men Who Have Sex with Men Using HIV Pre-Exposure Prophylaxis in Brazil: A Cross-Sectional Study

**DOI:** 10.3390/vaccines14010092

**Published:** 2026-01-18

**Authors:** Alvaro Francisco Lopes de Sousa, Lariane Angel Cepas, Isadora Silva de Carvalho, Caíque Jordan Nunes Ribeiro, Guilherme Reis de Santana Santos, Jean Carlos Soares da Silva, Talia Gomes Luz, Ruan Nilton Rodrigues Melo, Lucas Brandão dos Santos, Julia Bellini Sorrente, Gabriela Amanda Falsarella, Antonio Luis Ferreira Calaço, Ana Paula Morais Fernandes

**Affiliations:** 1 Campus de Três Lagoas, Universidade Federal de Mato Grosso do Sul, Três Lagoas 79070-900, MS, Brazil; 2 Public Health Research Centre, Comprehensive Health Research Center, CHRC, REAL, NOVA University Lisbon, 1050-091 Lisbon, Portugal; anapaula@eerp.usp.br; 3 Department of General and Specialized Nursing, Ribeirão Preto College of Nursing, University of São Paulo, Ribeirão Preto 14040-902 SP, Brazil; larianeangelcepas@usp.br (L.A.C.); isadoracarvalho@usp.br (I.S.d.C.); jeancarloseerp@usp.br (J.C.S.d.S.); taliagomesluz@usp.br (T.G.L.); ruan.melo@usp.br (R.N.R.M.); lucas.brandao.santos@alumni.usp.br (L.B.d.S.); julia.sorrente@usp.br (J.B.S.); gabrielafalsarella@usp.br (G.A.F.); antoniocalaco@usp.br (A.L.F.C.); 4 Department of Nursing, Federal University of Sergipe, Lagarto 49100-000, SE, Brazil; caiquejordan@academico.ufs.br (C.J.N.R.); guilhermereis22@academico.ufs.br (G.R.d.S.S.)

**Keywords:** human papillomavirus, vaccine, men who have sex with men, HIV, PrEP, sexually transmitted infections, vaccination coverage, Brazil

## Abstract

**Background**: Men who have sex with men (MSM) using HIV pre-exposure prophylaxis (PrEP) experience a high burden of human papillomavirus (HPV) infection and related diseases, yet data on HPV vaccination among this group in Brazil remain limited. **Aims**: The aims of this study were to estimate the prevalence of complete HPV vaccination and to identify factors associated with vaccination completion among MSM using PrEP in Brazil. **Methods**: We conducted a cross-sectional online survey between May and September 2025 among MSM aged ≥18 years, residing in Brazil and currently using oral PrEP. Participants were recruited through virtual snowball sampling and targeted advertisements on social media and a gay geosocial networking application. Data were collected using a structured, self-administered questionnaire hosted on REDCap^®^. Complete HPV vaccination was defined as self-reported receipt of all doses recommended according to the participant’s age and clinical condition. Sociodemographic characteristics, relationship patterns, sexual behaviors, lubricant use during sexual activity, and history of sexually transmitted infections (STIs) were assessed. Adjusted prevalence ratios (aPRs) and 95% confidence intervals (95% CIs) were estimated using Poisson regression with robust (sandwich) variance. **Results**: A total of 872 MSM using PrEP were included, of whom 59.4% reported complete HPV vaccination. In adjusted analyses, complete vaccination was more frequent among participants reporting both steady and casual partners (aPR = 1.90; 95% CI: 1.36–2.65) or only casual partners (aPR = 1.72; 95% CI: 1.24–2.39), those reporting lubricant use during sexual activity (aPR = 1.41; 95% CI: 1.23–1.61), and those with a diagnosis of chlamydia and/or gonorrhea in the previous 12 months (aPR = 1.22; 95% CI: 1.08–1.36). **Conclusions**: Although HPV vaccination coverage among MSM using PrEP in Brazil is higher than that reported for MSM in general, it remains incomplete in a population with regular contact with specialized health services. Integrating systematic assessment and delivery of HPV vaccination into PrEP care may help increase vaccination completion and reduce missed opportunities for prevention.

## 1. Introduction

Human papillomavirus (HPV) is one of the most common sexually transmitted infections (STIs) worldwide, with more than 200 identified genotypes, at least 12 of which are considered high-risk because of their oncogenic potential, particularly types 16 and 18 [1]. Persistent infection with these genotypes is causally associated with cervical cancer and with a substantial proportion of cancers of the anus, penis, vulva, vagina, and oropharynx [2,3]. It is estimated that approximately 4.5% of all cancers worldwide, corresponding to about 630,000 new cases per year, are attributable to HPV, with a disproportionately higher burden in low- and middle-income countries [2,4]. HPV therefore represents a major public health problem, the prevention of which necessarily relies on combined strategies of vaccination, screening, and reduction in social and behavioral vulnerabilities.

Genital HPV infection is generally asymptomatic and self-limited, but a proportion of infections persists and may progress to high-grade intraepithelial lesions and cancer over years or decades [1,5]. Most sexually active individuals will be exposed to the virus during their lifetime, with point prevalence of genital HPV infection often exceeding 30% among women and young men [5,6]. Although cervical cancer remains the most emblematic outcome of HPV infection, there is growing recognition of the impact of the virus on anogenital and oropharyngeal neoplasms in men, who already account for about 30% of HPV-related cancer cases in different settings [2,7].

In Brazil, the burden of HPV-associated diseases is substantial and characterized by marked regional inequalities. The Brazilian National Cancer Institute (INCA) estimates that for each year of the 2023–2025 triennium there will be about 17,010 new cases of cervical cancer, with a crude incidence rate of 15.4 cases per 100,000 women, making it the second most frequent cancer in the North and Northeast regions [8]. Data from the Brazil country report of the Catalan Institute of Oncology (ICO)/International Agency for Research on Cancer (IARC) also indicate a non-negligible incidence of anal, penile, and oropharyngeal cancers attributable to HPV, especially among men [9]. In terms of infection, the POP-Brasil study, conducted in a national sample of adolescents and young adults (16–25 years) who use the Brazilian Unified Health System (SUS in Portuguese), identified a prevalence of 53.5% for any HPV type and 35.2% for high-risk HPV, with a similar pattern between men and women, although with differences in genotype distribution [10]. These findings confirm the high circulation of the virus in the young Brazilian population and reinforce the need for targeted prevention strategies.

Among men specifically, a recent meta-analysis estimates a global prevalence of genital HPV infection of around one third of the male population, with a higher frequency of high-risk viral types in younger age groups and in groups with greater sexual exposure [6]. Among men who have sex with men (MSM), the HPV burden is even higher, particularly in the anal canal. Systematic reviews and meta-analyses indicate a prevalence of high-risk anal HPV exceeding 70% among MSM living with human immunodeficiency virus (HIV) and around 35–40% among HIV-negative MSM, as well as anal cancer incidence that can exceed 40 cases per 100,000 person-years in MSM with HIV, values comparable to or higher than those observed for cervical cancer before the introduction of mass vaccination [11,12,13]. Such evidence establishes MSM as a priority group for preventive interventions based on HPV vaccination and surveillance of anogenital lesions.

Studies of MSM in different countries also show a high frequency of sexually transmitted coinfections, multiple partners, inconsistent condom use, and substance use in sexual contexts, factors that increase both the likelihood of acquiring HPV and the persistence of infection [13,14]. Among users of human immunodeficiency virus pre-exposure prophylaxis (PrEP), a strategy widely targeted to MSM and other key populations, the prevalence of anal HPV infection and precursor lesions is also very high. Cohorts of MSM using PrEP in Europe and Latin America report prevalence of high-risk anal HPV that frequently exceeds 70%, with infection by multiple genotypes and a high occurrence of concurrent STIs [15,16]. In a study of MSM and transgender women on PrEP in Brazil, HPV was the most frequent infection, affecting about 70% of participants, illustrating the magnitude of vulnerability in this group [17].

In Brazil, daily oral PrEP has been available in the SUS since 2017 for people aged 15 years and older, with an emphasis on gay and other MSM, transgender people, sex workers, and people who use alcohol and other drugs, among other groups at increased risk for HIV infection [18]. The implementation of PrEP has created a new interface between key populations and health services, with regular visits, periodic testing for HIV and other STIs, and opportunities for health counseling. At the same time, analyses of morbidity profiles in these users show a substantial burden of bacterial and viral STIs, including HPV, prompting international discussions about integrating HPV vaccination into routine care in PrEP services [19,20].

In this context, HPV vaccination has emerged as a central pillar of the primary prevention of HPV-associated cancers. The quadrivalent HPV vaccine (HPV4), which protects against types 6, 11, 16, and 18, was incorporated into the Brazilian National Immunization Program (PNI) in 2014, initially for female adolescents aged 11 to 13 years, with the goal of reducing the incidence of cervical cancer in the country [21,22]. In subsequent years, the age range was adjusted to girls aged 9 to 14 years and, beginning in 2017, the PNI also began offering the vaccine to boys, initially aged 12 to 13 years and later expanded to 11 to 14 years, recognizing the role of HPV in anogenital and oropharyngeal cancers in men and the importance of herd immunity [23,24]. In parallel, the vaccine began to be recommended in a three-dose schedule for special groups aged 9 to 45 years, such as people living with HIV/AIDS, transplant recipients, patients with cancer, individuals with primary immunodeficiencies, and victims of sexual violence [25,26].

More recently, based on new evidence on the effectiveness of a single dose in adolescents and on data documenting the high burden of HPV infection and anal cancer in MSM, the Ministry of Health updated the national recommendations. In 2024, a single-dose schedule was adopted for immunocompetent adolescents aged 9 to 14 years and, through a Joint Technical Note, HPV4 vaccination was extended to PrEP users aged 15 to 45 years as a special strategy of the PNI [27,28]. This decision took into account, among other factors, the high prevalence of HPV infection and anal lesions in MSM using PrEP, the efficacy of the vaccine in preventing infection and anal intraepithelial neoplasia, and the opportunity to offer immunization in a context of regular follow-up, in which users already attend services semiannually or quarterly [15,16,28,29,30].

Despite this programmatic advance, important gaps remain regarding the vaccination coverage actually achieved among MSM using PrEP in Brazil and the individual, behavioral, and service organization factors associated with vaccination. Understanding how sociodemographic variables, prior experiences with STIs, PrEP use trajectories, access to health services, and risk perception relate to acceptance and receipt of the HPV vaccine in this group is crucial to guide active outreach strategies, health education, and integration among the components of combination prevention. In this context, investigating factors associated with HPV vaccination among MSM using PrEP in Brazil is essential to enhance the impact of recent PNI policies, reduce health inequities, and contribute to the prevention of anogenital cancers and other HPV-related diseases in a historically marginalized population.

On this basis, the aim of this study was to estimate the prevalence of HPV vaccination and to identify factors associated with vaccination among MSM using HIV PrEP who receive care in public health services in Brazil.

## 2. Materials and Methods

### 2.1. Study Design and Setting

This study is part of the PEG@ÇÃO project, an online study conducted throughout the Brazilian territory and coordinated by the Federal University of São Paulo. We present data from an analytical cross-sectional survey conducted between May and September 2025. The reporting of this study follows the STROBE (Strengthening the Reporting of Observational Studies in Epidemiology) statement for cross-sectional studies and the CHERRIES (Checklist for Reporting Results of Internet E-Surveys) guidelines.

### 2.2. Study Population and Eligibility Criteria

Individuals were eligible if they simultaneously met the following criteria: being 18 years of age or older; currently using oral PrEP provided by the SUS at the time of data collection; reporting sexual activity with men in the previous 12 months, regardless of gender identity or sexual orientation; being able to read and understand Portuguese; and agreeing to participate in the study by providing electronic consent.

The planned sample size was estimated based on an expected prevalence of complete HPV vaccination of approximately 50%, assuming a 95% confidence level and a margin of error of 3.5 percentage points, which resulted in a minimum required sample of approximately 784 participants. This conservative prevalence estimate was chosen to maximize sample size in the absence of prior national estimates for MSM using PrEP. Recruitment and paid dissemination were maintained until the planned sample size was reached and slightly exceeded, resulting in a final analytical sample of 872 participants.

### 2.3. Data Collection and Instrument

Data were collected through an online survey using a structured, self-administered questionnaire hosted on the REDCap^®^ platform (Vanderbilt University, Nashville, TN, USA). Access to the questionnaire occurred through a unique link that directed participants to the study home page, where the study objectives, ethical aspects, and the electronic informed consent form were presented; only after explicit agreement could the participant proceed to the questions. Access to the questionnaire required active electronic consent, and only participants who confirmed eligibility criteria were allowed to proceed. The REDCap^®^ survey was configured to prevent multiple submissions from the same device or browser session. No incentives were offered for participation, and records were reviewed for completion time and internal consistency prior to analysis.

To recruit participants, we used chain-referral (snowball) sampling adapted to the virtual environment, a strategy appropriate for hard-to-reach populations such as MSM using PrEP. Initially, the research team identified eligible participants in their contact networks and in previous initiatives related to PrEP and MSM health; these participants received the study link by digital means (messaging apps, email, social media) and were invited to share it with other people who were also MSM, living in Brazil, and using PrEP. Each new participant, upon completing the questionnaire, was likewise encouraged to pass the link on to their own network of contacts, thereby expanding the reach of the survey in a chain-like fashion.

In parallel, we conducted targeted dissemination on social media, specifically Facebook^®^ and Instagram^®^ (Meta Platforms Inc., Menlo Park, CA, USA). The team created standardized communication materials (digital cards) and a pinned post on the project page containing concise information about this study, participation criteria, and an invitation to complete the questionnaire, always accompanied by the link to REDCap^®^. These posts were boosted (paid promotion) to reach users in all regions of Brazil, with targeting toward adult men interested in topics related to sexual health, HIV, and sexual diversity, and remained active throughout the data collection period until the planned sample size was reached.

Given the centrality of PrEP and the profile of the target population, we also used the Hornet (Hornet Networks Inc., New York, NY, USA) platform, which is widely used by MSM in Brazil. We produced advertisements and invitations adapted to the platform layout, disseminated using geolocation tools that allowed for targeting of different regions of the country. The choice of Hornet leveraged the familiarity and comfort many MSM have with this digital environment, frequently used for social interaction and partner seeking, which potentially increases the response rate and the geographic diversity of the sample.

All invitations used neutral, respectful, and non-stigmatizing language, without reference to specific behaviors that could expose or embarrass potential participants. No personally identifiable information was collected through the outreach channels, and access to the questionnaire was anonymous. The instrument could be completed using smartphones, tablets, or computers, whether personal or shared, as long as the participant had internet access. All information used in the analysis was obtained exclusively through self-report by participants.

Due to the chain-referral design and the use of social media and geosocial networking platforms, it was not possible to determine the exact number of individuals exposed to the recruitment materials or the number of link impressions. However, all recruitment channels directed potential participants to a single survey link.

The instrument comprised different blocks of variables. The sociodemographic block included age (in completed years), self-reported race/skin color, sex assigned at birth, educational attainment, monthly household income in minimum-wage categories, main occupation, presence of any disability, and religious belief. These variables were used to characterize the social and economic profile of MSM using PrEP.

The block on relationships and sexual partnerships covered relationship status (no relationship, exclusive relationship, non-exclusive relationship), predominant type of sexual partnership (only steady partner, only casual partners, a combination of steady and casual partners, other), and use of dating apps to find sexual partners (yes/no).

The block on prevention practices and STI history assessed condom use during sexual intercourse, use of lubricating gel during sexual activity, primarily for comfort and facilitation of sexual practices, prior use of HIV post-exposure prophylaxis (PEP) at any point in life and specifically in the previous 12 months, as well as awareness and use of doxycycline post-exposure prophylaxis (doxy-PEP) in the previous 12 months. This same block included questions about physician-diagnosed STI in the 12 months preceding the survey, including gonorrhea and/or chlamydia, syphilis, mpox (human monkeypox), and hepatitis B and C.

Sexual practices were investigated in a specific block, considering behaviors associated with a higher risk of exposure to HPV and other STIs. Participants were asked about double penetration, fisting, footing, cruising, group sex, and bareback sex (intentional condomless anal sex). We also investigated the use of psychoactive substances in sexual contexts in the previous 12 months, including episodes of combining drug use and sex (chemsex), according to the participant’s own perception. All this information was obtained through closed-ended questions with dichotomous (yes/no) or predefined categorical response options.

Questionnaires with missing information on the primary outcome (HPV vaccination status) or presenting substantial internal inconsistencies were excluded prior to analysis. The final analytical sample comprised 872 participants.

### 2.4. Outcome

The outcome of interest was complete HPV vaccination status. Vaccination was assessed by self-report using a structured set of questions that investigated: (a) whether the participant had ever received the HPV vaccine; (b) the number of doses received; and (c) whether the recommended vaccination schedule had been completed. When answering these questions, participants were explicitly instructed to consult their vaccination records, including the Brazilian vaccination card (caderneta de vacinação) or official electronic immunization records available through national digital health platforms, when accessible.

Complete HPV vaccination was operationalized according to the recommendations of the Brazilian National Immunization Program (PNI) that were in force at the time each participant would have been eligible for vaccination, taking into account age and clinical condition. For immunocompetent individuals, completion was defined as receipt of the full age-appropriate schedule, including two doses for those vaccinated between 9 and 14 years under earlier PNI recommendations, or a single dose for adolescents covered by the updated 2024 policy. For immunocompromised individuals—including people living with HIV, transplant recipients, and other conditions defined by the PNI—completion was defined as receipt of a three-dose schedule, regardless of age at vaccination.

Following the 2024 Ministry of Health technical notes, users of HIV pre-exposure prophylaxis (PrEP) aged 15–45 years are classified as a priority group for HPV vaccination and are recommended to receive the quadrivalent HPV vaccine (HPV4). In this study, complete vaccination for PrEP users was defined as self-report of completion of the HPV vaccination schedule recommended by the PNI for this group at the time of vaccination, irrespective of whether vaccination occurred before or after PrEP initiation.

Participants who reported never having received the HPV vaccine, those who reported receiving fewer doses than recommended for their age or clinical condition, and those who were unable to reliably report the number of doses received or whether the vaccination schedule had been completed were conservatively classified as not having completed vaccination.

The outcome variable was therefore operationalized as binary (complete vaccination versus incomplete or no vaccination). The prevalence of complete HPV vaccination was estimated as the proportion of participants classified as having completed the recommended schedule relative to the total sample of men who have sex with men using PrEP.

### 2.5. Independent Variables

Independent variables included sociodemographic characteristics (age, race/skin color, sex assigned at birth, education, income, occupation, disability, religion); relational characteristics and type of sexual partnership (relationship status, predominant type of partnership, use of dating apps); prevention practices (condom use, use of lubricating gel, use of HIV post-exposure prophylaxis [PEP] ever and in the previous 12 months, awareness and use of doxycycline post-exposure prophylaxis [doxy-PEP]); history of STIs in the previous 12 months (gonorrhea/chlamydia, syphilis, mpox, hepatitis B and C); and sexual practices and substance use in sexual contexts (double penetration, fisting, footing, cruising, group sex, bareback sex, and use of psychoactive substances during sex). Selection of these variables was guided by theoretical plausibility and prior evidence on factors associated with vaccination and vulnerability to HPV among MSM using PrEP.

### 2.6. Data Analysis

Data collected in REDCap^®^ were exported in a compatible format, organized in a Microsoft Excel spreadsheet, and subsequently imported into the Statistical Package for the Social Sciences (SPSS), version 27.0 (SPSS Inc., Chicago, IL, USA; IBM Corp., Armonk, NY, USA), for statistical analysis.

Descriptive analyses were performed to summarize the study variables using absolute and relative frequencies. The prevalence of complete HPV vaccination was calculated, and row percentages were presented for each category of the independent variables. Percentages in the total column were calculated using the final analytical sample (n = 872) as the denominator.

Bivariate analyses were conducted using Pearson’s chi-squared test to explore associations between independent variables and complete HPV vaccination. Variables with a *p*-value < 0.20 in the bivariate analysis were considered eligible for inclusion in the multivariable model.

To estimate associations between explanatory variables and the outcome, prevalence ratios (PRs) and 95% confidence intervals (95% CIs) were calculated using Poisson regression models with a log-link function and robust (sandwich) variance estimation. This approach was chosen because the outcome was common (>10%), and the use of odds ratios from logistic regression could overestimate the magnitude of associations. Robust standard errors were applied to account for variance misspecification inherent to the use of Poisson models with binary outcomes.

Adjusted prevalence ratios (aPRs) and their respective 95% CIs were reported as measures of association. Multicollinearity among variables included in the multivariable model was assessed using tolerance values and the variance inflation factor (VIF).

Analyses were conducted using a complete-case approach. Participants with missing information on the primary outcome (HPV vaccination status) were excluded prior to analysis, as described in the data collection procedures. Missing data for independent variables were infrequent and handled by listwise deletion in multivariable analyses.

The final multivariable model was defined based on a combination of statistical criteria and theoretical relevance. Variables eligible according to the bivariate screening were retained if they demonstrated conceptual plausibility and contributed meaningfully to the model, resulting in a parsimonious final model including factors independently associated with complete HPV vaccination. Statistical significance was assessed using the Wald test, adopting a two-sided significance level of 5%.

### 2.7. Ethical Aspects

This study followed the ethical guidelines of National Health Council Resolution No. 466/2012 and was approved by the Research Ethics Committee of a public teaching and/or health institution (CAAE#85545824.2.0000.5393). Participation was voluntary and without any form of remuneration. Before accessing the REDCap^®^ questionnaire, potential participants were directed to a page containing the electronic informed consent form; only those who explicitly agreed proceeded to complete the survey. No nominal identifying information was collected, and the databases were stored in a secure environment with restricted access to the research team, ensuring participant confidentiality and anonymity.

## 3. Results

The final sample consisted of 872 MSM using PrEP, predominantly self-identified as White (n = 486; 55.73%), with male sex assigned at birth (n = 867; 99.43%), a monthly income between one and five times the minimum wage (n = 561; 64.33%), a main occupation in formal or salaried employment (n = 455; 52.18%), higher education or postgraduate schooling (n = 731; 83.83%), and a religious belief (n = 512; 58.72%). The prevalence of the outcome of interest (having been vaccinated against HPV) was 59.4% (Table 1).

Regarding variables related to romantic and sexual relationships, most participants reported being single (n = 624; 71.56%), having casual sexual partners (n = 755; 86.58%), and using dating apps (n = 802; 91.97%). Condom use (n = 538; 61.70%) and use of lubricating gel (n = 545; 62.50%) were the most commonly reported STI prevention methods.

A minority of participants reported having used PEP (n = 184; 21.20%) and doxy-PEP (n = 324; 37.16%) in the previous 12 months, as well as having received, in the same period, a diagnosis of gonorrhea/chlamydia (n = 201; 23.05%), mpox (n = 10; 1.15%), syphilis (n = 221; 25.34%), hepatitis B (n = 2; 0.230%), and hepatitis C (n = 8; 0.928%).

With regard to sexual practices, a minority reported engaging in double penetration (n = 129; 14.79%), fisting (n = 63; 7.22%), footing (n = 12; 1.38%), cruising (n = 232; 26.61%), and group sex (n = 303; 34.75%). By contrast, approximately half of the sample reported practicing bareback sex (n = 439; 50.34%) and having used some type of psychoactive substance during sex in the previous 12 months (n = 450; 51.61%) (Table 2).

A multivariable model was fitted to test the 21 variables eligible according to the statistical criterion (*p*-value < 0.20). Of these, three remained significant in the final model and were associated with a higher prevalence of HPV vaccination. Having both steady and casual sexual partners was associated with a 90% higher prevalence of the outcome (aPR = 1.90; 95%CI: 1.36 to 2.65), while having only casual sexual partners was associated with a 72% higher prevalence (aPR = 1.72; 95%CI: 1.24 to 2.39). Lubricant use during sexual activity was more frequently reported among participants with complete HPV vaccination (aPR = 1.41; 95%CI: 1.23 to 1.61), whereas having been diagnosed with chlamydia and/or gonorrhea in the previous 12 months was associated with a 22% higher prevalence of the outcome (aPR = 1.22; 95%CI: 1.08 to 1.36) (Table 3).

## 4. Discussion

The prevalence of complete HPV vaccination in this study was 59.4% among MSM using PrEP in Brazil. This is a relatively high level of coverage compared with studies conducted with MSM recruited in community settings or sexual health services, in which the proportion of vaccinated individuals usually ranges from 20% to 40%, often considering only “at least one dose” rather than completion of the full schedule. In an online survey of MSM in Ontario, Canada, for example, only 27.3% reported having received the HPV vaccine [31].

In the United States, analyses from surveillance systems indicate that only 17.9% of adult MSM and 32.8% of those aged 18–26 years reported having received any dose of the vaccine in 2017 [32]. In this context, the proportion of complete schedules found in this study among MSM using PrEP in Brazil suggests that this population, which is followed regularly in specialized services, may be benefiting from additional opportunities for vaccination compared with MSM who do not attend these services.

On the other hand, when compared with specific settings where integration between PrEP and vaccination is more structured, the coverage observed here still reveals important gaps. In a French study of MSM using PrEP, conducted with a mixed-methods approach, HPV vaccination coverage (at least one dose) among those eligible for reimbursement reached 71.2% [33]. Similarly, data from the community-based RiiSH-Mpox survey in the United Kingdom, conducted with gay, bisexual, and other MSM, indicated approximately 65% coverage for HPV among those eligible, with schedule completion rates above 75% for different vaccines [34].

Thus, although the coverage observed among MSM using PrEP in Brazil is higher than that reported in many studies of MSM in general, it still falls short of the level considered ideal for this high-risk population and of what is observed in settings where HPV vaccination is fully integrated into PrEP care, with systematic offering of the vaccine at virtually every visit and strong follow-up to ensure schedule completion.

Interpretation of these findings becomes even more relevant when considering the risk of HPV infection and anal neoplasia in MSM, particularly those with higher-risk sexual practices or living with HIV. Studies document prevalence of high-risk anal HPV infection exceeding 80% among MSM attending specialized services, as well as high rates of oral infection in specific subgroups [35,36]. This scenario reinforces the strategic importance of leveraging the regular follow-up of PrEP users to systematically offer the complete HPV vaccination schedule, similar to what has already been proposed for other vaccines targeted to MSM, such as hepatitis A and B.

The factors associated with complete vaccination identified in this study underscore the central role of these individuals being “anchored” in sexual health services and in a culture of combination prevention. In our study, MSM with both steady and casual partners, as well as those with only casual partners, had a higher prevalence of complete vaccination. These findings are consistent with international evidence showing a greater likelihood of vaccination among MSM who have a higher number of partners, more frequent STI testing, and more regular use of sexual health services [31,32,34]. In other words, individuals with more active sexual lives and greater risk perception seem to be more responsive to vaccine recommendations and more frequently exposed to vaccination opportunities during routine visits.

The observed association between lubricant use during sexual activity and a higher prevalence of complete HPV vaccination suggests that HPV immunization is embedded within a broader constellation of sexual health practices. Studies among MSM and PrEP users indicate that individuals who report multiple sexual health–related practices—such as condom use, PrEP, PEP, frequent STI testing, and, in some settings, doxycycline post-exposure prophylaxis—also tend to show greater adherence to vaccination schedules [34,37]. In this study, lubricant use should be interpreted not as a preventive measure against STIs, but rather as a marker of access to sexual health resources and closer engagement with specialized prevention services, where opportunities for HPV vaccination are more readily available.

Another relevant finding is the association between a recent history of gonorrhea and/or chlamydia and complete HPV vaccination. This pattern has also been observed in studies of MSM using PrEP and other at-risk populations, in which the presence of STIs at the first visit or during follow-up is positively correlated with a higher likelihood of being adequately vaccinated against HPV and hepatitis [34,38]. This association may reflect two complementary mechanisms: on the one hand, greater clinical vigilance after STI episodes, during which providers take the opportunity to update the vaccination schedule; on the other, users’ own perception of vulnerability, which makes them more receptive to vaccination recommendations after experiencing an episode of infection.

At the same time, the persistence of 40.6% of MSM without a complete vaccination schedule, even in a group with regular contact with PrEP services, reveals a pattern of “missed opportunities” widely described in the literature. Review studies emphasize that integration between PrEP and vaccination remains incomplete in many settings, whether because of failures in the systematic offering of vaccines, communication barriers, or the perception that vaccination is not a priority during PrEP visits [19,39]. In addition, qualitative studies with PrEP users show that many recognize the importance of the vaccine but report not having received clear information, not recalling having been invited to be vaccinated, or not knowing whether they completed the schedule [19,20,39].

The results presented here therefore reinforce the need to institutionalize HPV vaccination as an explicit component of PrEP care, with protocols that include checking vaccination history and prescribing and scheduling subsequent doses as part of routine visits. International experiences suggest that the formal inclusion of vaccination targets in PrEP guidelines, combined with training providers to address sexuality and prevention without stigma, helps increase vaccination coverage among MSM [20,33].

Interpretation of the findings should take into account that the sample is composed predominantly of MSM with high educational attainment, intermediate or high income, and strong engagement with health services, which tends to maximize the likelihood of vaccination. Even so, a substantial proportion remains without a complete schedule, suggesting that barriers related to systematic vaccine offering, communication, and organization of care persist even in contexts with greater access. In other MSM populations with lower educational levels, outside major urban centers, or not linked to PrEP, it is plausible that coverage is even lower, as suggested by studies with MSM in community-based samples in different countries [31,32].

### 4.1. Implications for Public Policy Formulation

The evidence from this study reinforces the notion that HPV vaccination should no longer be treated as an “optional” offer and instead become a core component of PrEP care. The prevalence of complete vaccination (59.4%) among MSM using PrEP is higher than that reported in many surveys of MSM in general, but remains below what would be expected for a group at very high risk and in regular follow-up in specialized services. Studies in other settings show that PrEP initiation and follow-up visits are privileged, but often missed, opportunities for updating vaccination, including for hepatitis A and HPV [20,38,40]. Our estimates support the development of national protocols that explicitly include systematic verification of HPV vaccination status at all stages of PrEP care, with proactive offering of the vaccine and scheduling of subsequent doses as a minimum standard for quality of care.

The factors associated with complete vaccination identified here also point to concrete directions for active outreach policies. MSM with multiple partners, a recent history of chlamydia/gonorrhea, and greater engagement in prevention strategies (such as the use of lubricating gel) were precisely those with a higher likelihood of being vaccinated, a pattern similar to that described in studies of young sexual minorities and PrEP users in other countries [38,41,42]. This suggests that teams are already partially using STI-related visits and contexts of heightened risk perception to propose vaccination. Public policy can build on this logic by establishing each PrEP initiation visit, every episode of bacterial STI, and every periodic review of the combination prevention regimen as “mandatory” points for offering HPV vaccination, with specific coverage targets for MSM using PrEP and routine monitoring of missed opportunities [20,39]. Finally, recognition of the effectiveness of vaccination in preventing penile and anal HPV infections in MSM and transgender women [43], together with the high level of exposure observed in our sample, indicates that expanding HPV coverage in this group is a cost-effective strategy for preventing anal cancer and other HPV-related neoplasms. From a public policy standpoint, this implies integrating information systems (PrEP and immunization) to identify pockets of low coverage, investing in ongoing training on HPV and LGBTQIA+ health for professionals in STI/HIV services, and using PrEP as an expanded platform for sexual health—coordinating HPV vaccination, vaccines for hepatitis and mpox, and other harm reduction interventions within a single care model [20,38].

### 4.2. Limitations

Interpretation of this study’s findings should take into account several limitations. The cross-sectional design precludes establishing causal relationships between the factors analyzed and complete HPV vaccination. In addition, all information was obtained by self-report, including vaccination status, which may introduce recall bias. Participants were recruited through chain-referral sampling via social media and a geosocial app, resulting in a profile predominantly composed of White MSM with high educational attainment and strong linkage to PrEP services, which limits generalizability to MSM in contexts of greater social vulnerability or outside major urban centers. Finally, this study was conducted in a context of successive changes in HPV vaccination policies in Brazil since vaccine implementation, with modifications in age ranges, priority groups, and the recent inclusion of PrEP users. Although we applied a conservative classification rule, changes in HPV vaccination schedules over time and reliance on self-reported dose counts may have led to non-differential misclassification, which would tend to bias associations toward the null. Some participants may have been vaccinated during earlier phases of the policy (for example, in adolescence or based on other eligibility criteria), and it was not possible to accurately reconstruct the timing and context of vaccination. Thus, the observed coverage reflects the sum of different access trajectories and cannot be attributed exclusively to the current recommendation for vaccination among PrEP users.

## 5. Conclusions

In this study, we estimated that approximately six out of every ten MSM using PrEP in Brazil reported having completed the full HPV vaccination schedule, indicating higher coverage than that described for most men and MSM in the general population, but still insufficient for a group in regular follow-up at specialized services and with high exposure to STIs. This result indicates that HPV vaccination is indeed reaching a substantial proportion of MSM on PrEP, but also reveals a sizable contingent that remains without complete protection despite frequent contact with combination prevention services.

With regard to associated factors, complete HPV vaccination was more frequently reported among MSM who had casual sexual partners—particularly those with both steady and casual partners—among those who reported lubricant use during sexual activity, and among those with a recent diagnosis of chlamydia and/or gonorrhea. These findings suggest that vaccination is more concentrated among individuals with greater engagement in prevention practices, higher risk perception, and more frequent use of sexual health services, where opportunities to offer the vaccine are more common. At the same time, they indicate that MSM with patterns of lower perceived exposure or less use of sexual self-care strategies may be left at the margins of immunization efforts.

## Figures and Tables

**Table 1:** Sociodemographic characteristics of MSM using PrEP according to complete HPV vaccination status (n = 872).

Variables	Yes (n = 518)		No (n = 354)		Total (n = 872)		*p*-Value
	n	%	n	%	n	%	
Race							
White	308	63.4	178	36.6	486	55.73	0.002
Black	80	60.6	52	39.4	132	15.14	
Brown	120	50.4	118	49.6	238	27.29	
Yellow	8	88.9	1	11.1	9	1.03	
Indigenous	2	50.0	2	50.0	4	0.46	
Prefer not to answer	-	-	3	100.0	3	0.34	
Sex assigned at birth							
Male	516	59.5	351	40.5	867	99.43	0.376
Female	2	40.0	3	60.0	5	0.57	
Monthly income							
Up to 1 minimum wage	43	74.1	15	25.9	58	6.65	0.002
Between 1 and 3 minimum wages	156	52.2	143	47.8	299	34.29	
Between 4 and 5 minimum wages	173	66.0	89	34.0	262	30.05	
Between 6 and 8 minimum wages	63	52.9	56	47.1	119	13.65	
More than 9 minimum wages	71	62.3	43	37.7	114	13.07	
Prefer not to answer	12	60.0	8	40.0	20	2.29	
Student							
Yes	80	58.8	56	41.2	136	15.60	0.881
No	438	59.5	298	40.5	736	84.40	
Homemaker							
Yes	7	87.5	1	12.5	8	0.92	0.152
No	511	59.1	353	40.9	864	99.08	
Formal or salaried worker							
Yes	279	61.3	176	38.7	455	52.18	0.229
No	239	57.3	178	42.7	417	47.82	
Self-employed professional							
Yes	93	55.0	76	45.0	169	19.38	0.197
No	425	60.5	278	39.5	703	80.62	
Self-employed, freelancer, or occasional worker							
Yes	69	68.3	32	31.7	101	11.58	0.052
No	449	58.2	322	41.8	771	88.42	
Unemployed with government assistance							
Yes	5	55.6	4	44.4	9	1.03	0.813
No	513	59.4	350	40.6	863	98.97	
Unemployed without government assistance							
Yes	17	65.4	9	34.6	26	2.98	0.528
No	501	59.2	345	40.8	846	97.02	
Entrepreneur or business owner							
Yes	29	43.9	37	56.1	66	7.57	0.008
No	489	60.7	317	39.3	806	92.43	
Education							
Primary education	4	57.1	3	42.9	7	0.80	0.053
Secondary education	45	57.7	33	42.3	78	8.94	
Technical education	33	62.3	20	37.7	53	6.08	
Higher education	224	65.7	117	34.4	341	39.11	
Postgraduate education	210	53.8	180	46.2	390	44.72	
Prefer not to answer	2	66.7	1	33.3	3	0.34	
Has a disability							
Yes	9	36.0	16	64.0	25	2.87	0.012
No	509	60.2	336	39.8	845	96.90	
Prefer not to answer	-	-	2	100.0	2	0.23	
Religion							
Yes, practicing	119	45.9	140	54.1	259	29.70	<0.001
Yes, non-practicing	148	58.5	105	41.5	253	29.01	
None	245	70.8	101	29.2	346	39.68	
Prefer not to answer	6	42.9	8	57.1	14	1.61	

**Table 2:** Relational characteristics, sexual behaviors, use of prevention strategies, and STI history among MSM using PrEP according to complete HPV vaccination status (n = 872).

Variables	Yes (n = 518)		No (n = 354)		Total (n = 872)		*p*-Value
	n	%	n	%	n	%	
Current relationship status							
Yes, exclusive	26	55.3	21	44.7	47	5.39	<0.001
Yes, non-exclusive	142	71.7	56	28.3	198	22.71	
None	348	55.8	276	44.2	624	71.56	
Prefer not to answer	2	66.7	1	33.3	3	0.34	
Type of sexual partnership							
Steady partner	27	32.9	55	67.1	82	9.40	<0.001
Casual partner	271	60.1	180	39.9	451	51.72	
Steady + casual partners	194	63.8	110	36.2	304	34.86	
Other	5	41.7	7	58.3	12	1.38	
Prefer not to answer	21	91.3	2	8.7	23	2.64	
Uses dating apps							
Yes	469	58.5	333	41.5	802	91.97	0.060
No	49	70.0	21	30.0	70	8.03	
Uses condoms							
Yes	323	60.0	215	40.0	538	61.70	0.629
No	195	58.4	139	41.6	334	38.30	
Uses lubricating gel							
Yes	367	67.3	178	32.7	545	62.50	<0.001
No	151	46.2	176	53.8	327	37.50	
PEP (ever)							
Yes	42	55.3	34	44.7	76	8.72	0.442
No	476	59.8	320	40.2	796	91.28	
PEP use in the last 12 months							
Yes	67	36.4	117	63.6	184	21.10	<0.001
No	447	65.7	233	34.3	680	77.98	
Do not know	2	50.0	2	50.0	4	0.46	
Prefer not to answer	2	50.0	2	50.0	4	0.46	
Doxy-PEP use in the last 12 months							
Yes	216	66.7	108	33.3	324	37.16	0.007
No	244	55.3	197	44.7	441	50.57	
Do not know	57	53.8	49	46.2	106	12.16	
Prefer not to answer	1	100.0	-	-	1	0.11	
Diagnosed with gonorrhea and/or chlamydia in the last 12 months							
Yes	133	66.2	68	33.8	201	23.05	0.026
No	385	57.4	286	42.6	671	76.95	
Diagnosed with mpox in the last 12 months							
Yes	8	80.0	2	20.0	10	1.15	0.215
No	510	59.2	352	40.8	862	98.85	
Diagnosed with syphilis in the last 12 months							
Yes	150	67.9	71	32.1	221	25.34	0.003
No	368	56.5	283	43.5	651	74.66	
Diagnosed with hepatitis B in the last 12 months							
Yes	2	100.0	-	-	2	0.23	0.517
No	516	59.3	354	40.7	870	99.77	
Diagnosed with hepatitis C in the last 12 months							
Yes	5	62.5	3	37.5	8	0.92	1.000
No	513	59.4	351	40.6	864	99.08	
Practices double penetration							
Yes	86	66.7	43	33.3	129	14.79	0.069
No	432	58.1	311	41.9	743	85.21	
Practices fisting							
Yes	36	57.1	27	42.9	63	7.22	0.704
No	482	59.6	327	40.4	809	92.78	
Practices footing							
Yes	8	66.7	4	33.3	12	1.38	0.770
No	510	59.3	350	40.7	860	98.62	
Practices cruising							
Yes	150	64.7	82	35.3	232	26.61	0.057
No	368	57.5	272	42.5	640	73.39	
Practices bareback sex							
Yes	284	64.7	155	35.3	439	50.34	0.001
No	234	54.0	199	46.0	433	49.66	
Practices group sex							
Yes	187	61.7	116	38.3	303	34.75	0.310
No	331	58.2	238	41.8	569	65.25	
Practiced chemsex in the last 12 months							
Yes	284	63.1	166	36.9	450	51.61	0.013
No	219	54.8	181	45.3	400	45.87	

**Table 3:** Factors associated with complete HPV vaccination among MSM using PrEP in Brazil: Poisson regression model with robust variance (n = 872).

Variables	β	aPR	95% CI (Lower)	95% CI (Upper)	*p*-Value
Intercept	−1.351	0.26	0.19	0.36	<0.001
Has both steady and casual sexual partners	0.640	1.90	1.36	2.65	<0.001
Has only casual sexual partners	0.541	1.72	1.24	2.39	0.001
Uses lubricating gel during sexual activity	0.340	1.41	1.23	1.61	<0.001
Diagnosis of chlamydia and/or gonorrhea in the last 12 months	0.195	1.22	1.08	1.36	<0.001

## Data Availability

The data that support the findings of this study are available from the corresponding author upon reasonable request. Due to privacy protections and ethical restrictions related to sensitive sexual health information, the dataset is not publicly available. The aggregate results and analytical scripts may be shared upon justified request.

## Abbreviations

The following abbreviations are used in this manuscript:

HPV	Human papillomavirus
STI	Sexually transmitted infection
MSM	Men who have sex with men
HIV	Human immunodeficiency virus
AIDS	Acquired immunodeficiency syndrome
PrEP	Pre-exposure prophylaxis
PEP	Post-exposure prophylaxis
Doxy-PEP	Doxycycline post-exposure prophylaxis
SUS	Brazilian Unified Health System
INCA	Brazilian National Cancer Institute
ICO	Catalan Institute of Oncology
IARC	International Agency for Research on Cancer
PNI	Brazilian National Immunization Program
REDCap	Research Electronic Data Capture
SPSS	Statistical Package for the Social Sciences
aPR	Adjusted prevalence ratio
PR	Prevalence ratio
OR	Odds ratio
CI	Confidence interval
VIF	Variance inflation factor
AIC	Akaike Information Criterion
mpox	Human monkeypox
HBV	Hepatitis B virus
HCV	Hepatitis C virus
LGBTQIA+	Lesbian, gay, bisexual, transgender, queer, intersex, asexual, and other sexual and gender minorities
